# Dr. James Decker Spencer

**Published:** 1910-06

**Authors:** 


Dr. James Decker Spencer, of Watertown, N. Y., died at his home in that city May 5, 1910, aged 62 years. He graduated in medicine at Bellevue Hospital Medical College in 1870, and soon associated himself with his father in the practice of his profession. He was a member of the American Public Health Association of the New York Academy of Medicine, of the Medical Society of the County of Jefferson and of the Medical Society of the State of New York, of which he was president in 1897. Dr. Spencer for many years previous to his death, was regarded as the leading physician in the region of his residence. He was a leader in all the important affairs of his environment, a distinguished citizen, and an upright man. His loss will be keenly felt by all the people in that community.

